# Poliovirus immunity among adults in the Democratic Republic of the Congo: a cross-sectional serosurvey

**DOI:** 10.1186/s12879-021-06951-6

**Published:** 2022-01-05

**Authors:** Vivian H. Alfonso, Arie Voorman, Nicole A. Hoff, William C. Weldon, Sue Gerber, Adva Gadoth, Megan Halbrook, Amelia Goldsmith, Patrick Mukadi, Reena H. Doshi, Guillaume Ngoie-Mwamba, Trevon L. Fuller, Emile Okitolonda-Wemakoy, Jean-Jacques Muyembe-Tamfum, Anne W. Rimoin

**Affiliations:** 1 Department of Epidemiology, University of California, Los Angeles, USA; 2 McKing Consulting, Atlanta, GA USA; 3 Bill and Melinda Gates Foundation, Seattle, WA USA; 4 Division of Viral Diseases, National Center for Immunization and Respiratory Diseases, Centers for Disease Control and Prevention, Atlanta, GA USA; 5 National Institute for Biomedical Research (INRB), Kinshasa, Democratic Republic of the Congo; 6 Division of Global HIV and Tuberculosis, Center for Global Health, Centers for Disease Control and Prevention, Atlanta, GA USA; 7 Expanded Programme on Immunization, Kinshasa, Democratic Republic of the Congo; 8 Center for Tropical Research, Institute of the Environment and Sustainability, University of California, Los Angeles, USA; 9 Kinshasa School of Public Health, Kinshasa, Democratic Republic of the Congo

**Keywords:** Poliovirus, Polio, Immunization, Vaccine-preventable diseases, Adult immunity, Democratic Republic of the Congo, Demographic and Health Survey

## Abstract

**Background:**

Vaccination efforts to eradicate polio currently focus on children under 5 years of age, among whom most cases of poliomyelitis still occur. However, in the Democratic Republic of the Congo (DRC), an outbreak of wild poliovirus type 1 occurred in 2010–2011 in which 16% of cases occurred among adults; in a related outbreak in the neighboring Republic of Congo, 75% of cases occurred among the same adult age-group. Given that infected adults may transmit poliovirus, this study was designed to assess adult immunity against polioviruses.

**Methods:**

We assessed poliovirus seroprevalence using dried blood spots from 5,526 adults aged 15–59 years from the 2013–2014 Demographic and Health Survey in the DRC.

**Results:**

Among adults in the DRC, 74%, 72%, and 57% were seropositive for neutralizing antibodies for poliovirus types 1, 2, and 3, respectively. For all three serotypes, seroprevalence tended to be higher among older age groups, those living in households with more children, and among women.

**Conclusions:**

Protection against poliovirus is generally low among adults in the DRC, particularly for type 3 poliovirus. The lack of acquired immunity in adults suggests a potentially limited poliovirus circulation over the lifetime of those surveyed (spanning 1954 through 2014) and transmission of vaccine-derived poliovirus in this age group while underscoring the risk of these outbreaks among adults in the DRC.

**Supplementary Information:**

The online version contains supplementary material available at 10.1186/s12879-021-06951-6.

## Background

While no cases of wild poliovirus (WPV) have been detected in the Democratic Republic of the Congo (DRC) since 2011 [1], endemic transmission continues in Afghanistan, and Pakistan [2]. The elimination of WPV in the DRC was a gradual process: the last reported cases occurred in 1997, 2009 and 2011 for WPV serotypes 2, 3 and 1, respectively [3]. Furthermore, WPV type 2 has been declared globally eradicated, with the last case of paralytic polio from WPV2 observed in Uttar Pradesh, India in 1999 [4]. However, circulating vaccine-derived poliovirus (cVDPV) outbreaks remain a concern and, at the time of writing, there were 9 independent cVDPV outbreaks occurring in the DRC [5]. Until polio is eradicated and oral polio vaccine (OPV) withdrawn from use, the risk of outbreaks from WPV importation and emergence of cVDPV cases may persist due to sub-optimal vaccination coverage [6].

Prior to the initiation of routine and supplemental immunization programs, high rates of endemic infection among children resulted in infrequent paralytic poliomyelitis cases among adults in developing countries [7]. However, the introduction of routine immunization reduced poliovirus circulation and, as unvaccinated or inadequately vaccinated subgroups of children, outbreak-associated cases may occur among older children, adolescents and adults [7]. In the DRC, past outbreaks have affected adult populations and underscore the role and importance of adults in transmission, outbreaks, and efforts to eradicate the disease. In 2010 and 2011, respectively, there were WPV1 outbreaks of 100 cases (in 5 provinces) and 93 cases (in 6 provinces) in the DRC [3]. Among all cases with known ages, 5% in 2010 and 29% in 2011 were identified among individuals aged ≥ 15 years (age range 16–31 years). Prior to these outbreaks, nearly all documented WPV cases in the DRC had occurred in children < 15 years of age [8]. At the same time, the Republic of Congo experienced an outbreak of 445 polio cases, with nearly 75% occurring among adults [6]. During that outbreak, the median age of laboratory-confirmed and clinical polio cases was 20 years (range, < 1–63 years) and individuals 15 to 29 years of age had the highest incidence of all age groups. In response to the outbreaks, national and sub-regional supplemental immunization activities (SIAs) targeted the entire population, adapting strategies to reach adults. Furthermore, cVDPV2 cases have also been documented in adults: while the vast majority of the 315 cVDPV2 cases identified in Nigeria between 2005 and 2010 occurred in children between 18 and 36 months of age, cases were identified in adults up to 22 years of age [9].

Serological protection among adults is also related to transmissibility of wild and vaccine viruses [10, 11]. While the DRC Expanded Program on Immunization (EPI) was introduced in 1978, many adults in the current study were born when routine immunization coverage was low, and wild polioviruses were endemic. Historically, SIAs typically targeted children less than 5 years of age; however, during the 2010–2011 outbreak, all-age campaigns were conducted in certain provinces throughout the DRC, making some adults eligible for supplemental vaccination. This variation makes precise estimation of adult participation in transmission, or generalization of any such estimate, difficult. Since acute flaccid paralysis (AFP) monitoring is the cornerstone of polio surveillance, this estimation is further complicated by the fact that most infections present sub-clinically. Approximately 1 in 150 primary infections cause paralytic poliomyelitis; thus, reports of paralytic cases provide a vast underestimate of total viral spread [4].

Among adults who were not vaccinated, serological protection must be due to exposure to WPV, cVDPV, or through exposure to the Sabin live, attenuated oral poliovirus shed by a vaccine recipient. Individuals with serum immunity against polio are protected from developing paralysis; however, as intestinal immunity to poliovirus wanes over time, exposure to the virus may result in infection of the gut, resulting in shedding and exposure to others [12, 13]. This mechanism may be an underlying reason for the finding of a case–control study in Angola that identified travel of adult household members outside the province of residence as a risk factor for poliomyelitis in children [14]. In addition, oral polio vaccine (OPV) strains can protect contacts of vaccine recipients through environmental secondary immunization, increasing the reach and impact of the vaccine [15]. Since transmission and reversion of OPV can also facilitate emergence of cVDPV in low-immunity settings, it is important to better understand secondary spread of OPV strains among adult household and community contacts.

Given the important role adults may play in the transmission of poliovirus and the global effort to eradicate polio, understanding adult immunity against polioviruses could be useful in identifying high risk populations in the DRC [16]. In this paper, we describe results from a serosurvey conducted in collaboration with the 2013–2014 DRC Demographic and Health Survey (DHS) on serological protection from poliovirus types 1, 2, and 3 among adults (persons 15 years of age and older). Identifying potential high-risk areas with large numbers of susceptible adults is essential for planning, management and eradication efforts due to the risk of continued cVDPV emergence or importation, as well as importation of WPV.

## Methods

### Study population, design and sample split

The second Demographic and Health Survey (DHS) took place in the DRC from November 2013 to February 2014, at which time trivalent OPV (types 1, 2, and 3) was being utilized as a part of routine immunization (RI) activities in the DRC under WHO guidelines (inactivated poliovirus vaccine (IPV) and bivalent OPV [types 1 and 3] are currently used in the DRC as of 2020). Using a 2-stage stratified cluster design, the survey generates nationally representative data on population health and social indices, with GPS-identified cluster sites. Details on the sampling design and data collection procedures are described elsewhere [17]. During the survey, 18,827 women aged 15 to 49 in all selected households, and 8656 men aged 15 to 59 in half of selected households were successfully interviewed. All adults from households selected for the male survey were eligible for HIV testing using dried blood spots (DBS). We subsequently were granted access to a single spot from available remaining samples from the DBS card which contained a total of 5 blood spots, each containing 3 6 mm punches collected on a Whatman 903 filter paper card to test for serologic protection against poliovirus.

To ensure sufficient power for analysis, DBS samples were divided into two subsets: polio, presented in this paper, and measles, mumps, varicella zoster, rubella and tetanus (MMVRT) from multiplex testing. This sub-setting was done to maintain two representative samples of adult participants and of the population from which they were drawn. Within each sampling cluster of the DRC DHS survey design, available samples were sorted by sex and age. The youngest two individuals of the same sex were assigned to a unique pair identification number, then the next two, and so on. Where an odd number of males or females appeared in the cluster, the oldest person was treated as a singleton. Within sample pairs, each individual was assigned a random number between 0 and 1; the participant that was assigned 0 was tested for neutralizing antibodies against polio at the CDC, while the remaining individuals were assigned to be assessed for antibody response against MMVRT at the UCLA-DRC laboratory at the National Institute for Biomedical Research (INRB) in Kinshasa, DRC. Lastly, singletons were assigned with a 0.5 probability to either the polio or MMVRT testing groups. Among the available 17,420 DBS samples from those selected and eligible for HIV testing (850 participants did not have a corresponding DBS sample obtained), 8713 were sent to CDC, while 8706 were retained at the INRB. Assessment of demographics across sample subsets showed comparable distribution across province, age and sex (Additional file 1: Table S1).

### Laboratory analysis

Following the DHS protocol, DBS samples were collected in the field from participating adults, transported at room temperature to the DRC’s National AIDS Control Program national reference laboratory in Kinshasa, and stored at – 80 °C until testing. One spot (up to three 6 mm punches) per subject was shared with the UCLA-DRC program at the INRB for testing of VPDs as described above. DBS were shipped at − 20 °C to CDC to test for neutralizing antibodies against poliovirus serotypes 1, 2, and 3. As described elsewhere, we used the modified poliovirus microneutralization assay which measures the ability of antibodies eluted from DBS punches to block the infectivity of poliovirus in an in vitro cell culture system [18, 19]. Upon receipt at the laboratory, the specimens were logged, randomized, and three 6 mm punches were collected from each card to be processed using the low-volume polio neutralization assay in 384-well plates. A neutralizing antibody titer greater than or equal to 1:8 (3.0 log_2_) was used as the threshold of seropositivity [19–21].

### Statistical analysis

Among the 8713 adult samples received by CDC, 2941 (33.8%) could not be evaluated due to insufficient sample volume (at least three 6 mm punches) and 60 were indicated as missing during randomization. Of the 5712 DBS samples assessed for neutralizing antibodies against poliovirus types 1, 2, and 3, 5526 (96.7%) were successfully merged with DHS sociodemographic survey data (2974 women and 2552 men), among whom 5,381 (97.4%) had results for all three serotypes (145 samples were not evaluated for neutralizing antibodies for type 3 due to an error in an assay plate and insufficient sample to retest) (Additional file 1: Fig. S1). Male survey sample weights for pairs and singletons were doubled to account for the adult DHS sample split and applied in analyses to obtain a nationally representative estimate of adult polio serological response. We adjusted for the missing data by raking the survey weights so that the total weight in the sub-sample with serology results matched the full sample by province, sex, age group, and rural/urban area.

We estimated population immunity to each poliovirus serotype in weighted analyses by sex, age group, education level, wealth index, presence of children in household (overall and the presence of children under 5 years of age), residence (urban or rural), and province. For serologic analyses, neutralization titers were dichotomized as follows: titers < 3.0 log_2_ are seronegative, while ≥ 3.0 log_2_ are categorized as seropositive. Rao-Scott chi-square tests, which account for the DHS study design, were used to assess differences in seropositivity across demographic characteristics using p-value ≤ 0.05 to classify a result as statistically significant. All analyses were performed in R version 4.1.0.

### Ethical approvals

Study methods were carried out in accordance with relevant guidelines and regulations. All subjects provided informed consent prior to enrollment, survey completion, and biological sampling. For minors, informed consent was obtained from parents or legal guardians. Ethical approval for this study was obtained from Institutional Review Boards at the UCLA Fielding School of Public Health, the Kinshasa School of Public Health, and the US Centers for Disease Control and Prevention (CDC).

## Results

Among adults participating in the 2013–2014 DRC-DHS, 74%, 72%, and 57% were seropositive for neutralizing antibodies for type 1, type 2 and type 3, respectively (Table 1). While little difference was observed by sex for type 1, women had higher seroprevalence rates compared with men for types 2 and 3. Immunity was positively correlated between serotypes: overall, 40% of adults were seropositive for all three types of polio (46% for women and 33% for men), while only 9% were negative across all serotypes.

**Table 1:** Distribution of polio serostatus among adults in the DRC

Serotype	All adults	Male	Female
Type 1	74 (72–75)	71 (69–73)	76 (74–78)
Type 2	72 (70–73)	65 (62–68)	78 (75–80)
Type 3	57 (56–59)	50 (47–53)	64 (62–66)
Types 1, 2, and 3	40 (38–42)	33 (30–35)	46 (44–48)
Types 1 and 2	59 (57–61)	52 (50–55)	64 (62–67)
Types 1 and 3	47 (45–49)	41 (38–44)	52 (50–54)
Types 2 and 3	46 (44–48)	38 (35–41)	54 (51–56)
Type 1 only	8 (7–9)	10 (9–12)	5 (4–6)
Type 2 only	6 (5–7)	8 (6–10)	5 (4–6)
Type 3 only	4 (3–5)	4 (3–5)	4 (3–5)
All negative	9 (8–10)	12 (10–14)	7 (6–8)

This table describes percent of adults within each serologic category, and associated 95% confidence intervals (95% CI)

The relationship between seropositivity and sociodemographic characteristics varied by serotype (Table 2). We generally saw higher rates of seroprevalence among adults in households with more children. Calculated from the number of children in the household, we observed 73%, 68%, and 52% seroprevalence for type 1, 2 and 3, respectively, for those without children and 74%, 74%, and 58% seroprevalence for type 1, 2 and 3, respectively, for those with five or more children. As both children and adults took part in the DHS serosurvey, we conducted exploratory analysis of seropositivity among children [19] and adults within households. A significant intrahousehold relationship in neutralizing antibodies was identified for poliovirus type 2 only (see Additional file 1: Table S2). Seroprevalence was generally higher in older populations, while we did not see much variation in seroprevalence by wealth index, education, or residence (rural vs urban).

**Table 2:** Seroprevalence by sociodemographic characteristics among adults in DRC, 2013–2014

Variable	Level	Proportion	Type 1	Type 2	Type 3
Age^a^	15–19	17	69 (66–73)	70 (66–75)	55 (50–59)
Age	20–24	19	71 (67–75)	70 (66–73)	53 (49–57)
Age	25–29	17	71 (66–75)	71 (66–75)	59 (54–63)
Age	30–34	14	77 (73–81)	69 (64–73)	59 (54–64)
Age	35–39	11	76 (72–81)	76 (72–81)	57 (52–63)
Age	40–44	9	75 (69–81)	78 (72–83)	66 (60–71)
Age	44 +	14	80 (76–83)	73 (69–77)	59 (54–63)
Age	NA	NA	p = 0.0044	p = 0.069	p = 0.022
Children 0–14	None	10	73 (69–78)	68 (63–73)	52 (47–56)
Children 0–14	1-2	29	71 (68–74)	69 (66–72)	54 (50–58)
Children 0–14	3-4	47	75 (73–78)	73 (71–76)	61 (58–63)
Children 0–14	5 +	14	74 (69–78)	74 (71–78)	58 (54–63)
Children 0–14	NA	NA	p = 0.19	p = 0.047	p = 0.0029
Children 0–5	None	26	71 (68–74)	69 (65–72)	53 (50–57)
Children 0–5	1	26	75 (71–78)	73 (70–76)	58 (54–61)
Children 0–5	2	30	74 (71–77)	72 (69–75)	59 (56–62)
Children 0–5	3	14	76 (72–80)	75 (71–78)	62 (57–66)
Children 0–5	4 +	4	77 (69–84)	77 (70–84)	60 (50–70)
Children 0–5	NA	NA	p = 0.32	p = 0.079	p = 0.046
Education	No education, preschool	10	72 (68–77)	75 (70–81)	63 (57–69)
Education	Primary	31	72 (69–75)	73 (70–76)	58 (54–61)
Education	Secondary	53	74 (72–76)	70 (67–72)	56 (53–59)
Education	Higher	6	80 (73–87)	72 (66–79)	61 (54–68)
Education	NA	NA	p = 0.23	p = 0.13	p = 0.14
Sex	Male	47	71 (69–73)	65 (62–68)	50 (47–53)
Sex	Female	53	76 (74–78)	78 (75–80)	64 (62–66)
Sex	NA	NA	p = 0.0018	p = 2e−10	p = 3.2e−12
Type of residence	Urban	37	76 (73–79)	71 (69–74)	58 (55–61)
Type of residence	Rural	63	72 (70–75)	72 (69–74)	57 (55–60)
Type of residence	NA	NA	p = 0.082	p = 0.65	p = 0.74
Wealth	Poorest	18	72 (69–76)	72 (69–76)	59 (55–63)
Wealth	Poorer	19	73 (69–78)	71 (67–75)	54 (49–59)
Wealth	Middle	20	72 (68–76)	72 (69–76)	57 (53–61)
Wealth	Richer	20	69 (65–74)	69 (65–73)	57 (53–62)
Wealth	Richest	23	80 (76–83)	73 (70–76)	60 (56–64)
Wealth	NA	NA	p = 0.0014	p = 0.49	p = 0.37

Evidence for variation in seroprevalence given by Rao-Scott chi-square p-value

^a^Women included in DHS survey ranged from 15–49 years, while men ranged from 15–59 years

**Fig. 1:**
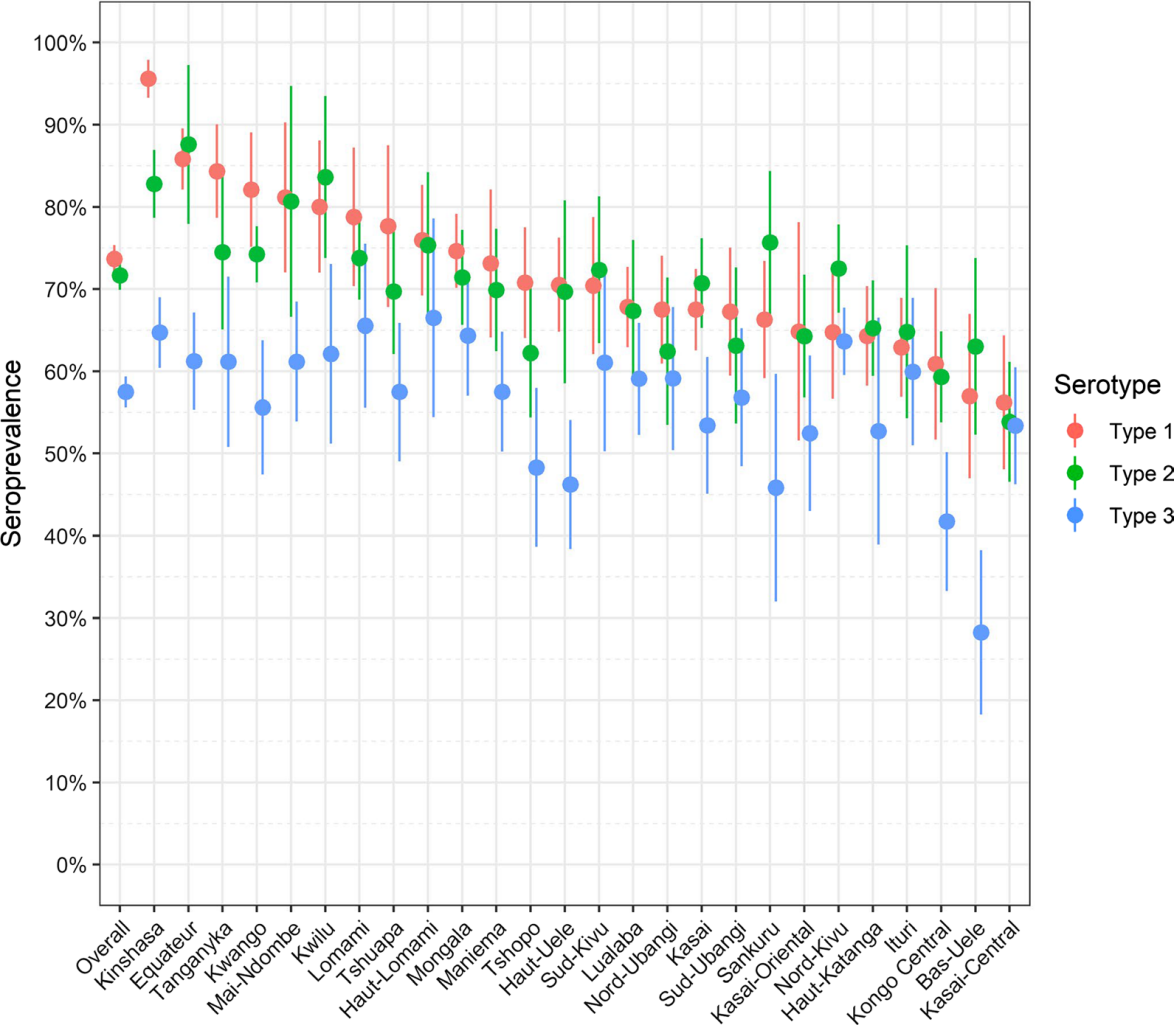
Distribution of Polio seropositivity (%) against Sabin types 1, 2 and 3 for the 26 provinces of the Democratic Republic of the Congo, DHS 2013–2014

While little difference was observed by the type of residence (urban or rural), seroprevalence varied widely across provinces (Fig. 1). Bas-Uele had the lowest seroprevalence in adults (28% for type 3), while the highest estimates of seropositivity were observed in Kinshasa for type 1 (96%), Equateur for type 2 (87%) and Haut-Lomami for type 3 (67%).

**Fig. 2:**
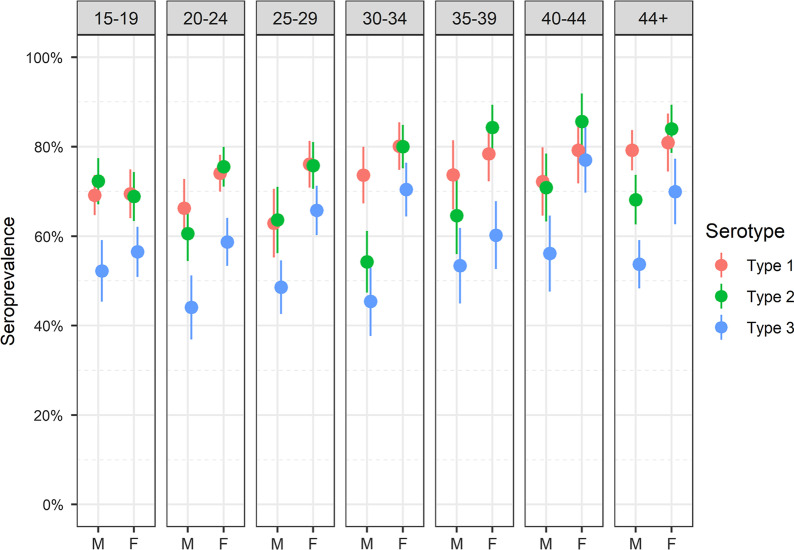
Distribution of Polio seropositivity (%) against Sabin type 1, 2 and 3 by age group in the Democratic Republic of the Congo DHS 2013–2014

As seroprevalence was higher among women overall, and this trend generally persisted within age groups as well (Fig. 2). Among adults older than 20 years of age, a higher proportion of women trended seropositive than men across all serotypes. The largest differences in seroprevalence estimates across sex were observed in the 30 to 34 year-olds, while those differences appeared smaller at the extremes of age. The largest gap in seropositivity between men and women occurred in those aged 25–29 for poliovirus type 1 (13%), in those 30–34 years old for type 2 (26%), and in those 30–34 years old for type 3 (25%) (Additional file 2, Additional file 3).

## Discussion

This study provides the first nationally representative estimates of neutralizing antibodies against poliovirus type 1, 2, and 3 in adults of the DRC. Among adults represented in the survey (men ages 15 to 59 years and women ages 15 to 49 years), an estimated 26%, 28% and 43% remain susceptible to poliovirus type 1, 2, and 3, respectively. As only 9% were seronegative for all three, the vast majority of adults exhibit some level of protection against one or more serotypes (approximately 40% of adults were positive for all three). Seropositivity was generally higher among women, those in households with more children, and among older age groups, and also varied by province.

Overall, we observed relatively low seroprevalence among adults across the country. In some areas, the low seroprevalence, particularly to type 3 poliovirus (57% overall), may indicate a limited viral circulation and/or possibly weak poliovirus transmissibility in the population, which permits individuals to escape infection through adulthood. In addition, this result may be influenced by the lower overall immunogenicity of poliovirus type 3 administered by trivalent OPV vaccine as compared to that from the type 1 and 2 components [22]. The buildup and geographic spread of susceptible populations may, in part, explain previous outbreaks that affected adult populations, a pattern more commonly observed in developed countries with high levels of sanitation [7]. In the context of the DRC, low seroprevalence could be instead explained by relatively low population movement and restricted geographic spread due to the difficulty of transportation between communities and historically rural character, rather than good sanitation and limited ability to spread to contacts of infected individuals. This is observed in some of the recent cVDPV2 outbreaks which are often regionally localized rather than country-wide. Future studies that include modeling of potential household secondary transmission of poliovirus from vaccinated children to adults could refine estimates of natural immunity and better clarify the extent of historical virus circulation.

In the present study, type 1 had the highest seroprevalence across all age and sex groups followed by types 2 and 3. Although the assay does not distinguish between neutralizing antibodies induced by exposure to WPV, OPV, or VDPV, this finding may reflect the atypical outbreak of wild poliovirus type 1 that occurred from 2010 to 2011 [3]. This outbreak affected a larger proportion of persons ≥ 15 years of age than expected in 4 of the 6 outbreak provinces, and triggered OPV campaigns covering all ages. We found the high seroprevalence for type 1 in Kinshasa (95%) and Kwilu province (80%) which had the highest proportion of cases that occurred among individuals 15 years of age and older during the outbreak (24% and 52%, respectively). Additionally, male sex was associated with case status among the adults from the 2010–2011 WPV1 outbreak, corresponding with our findings that there were larger immunity gaps among men. As the OPV virus is shed through fecal matter, the difference in seropositivity by sex may be a result of females taking on primary caregiver roles for infants and young children receiving OPV.

Similar to the serosurvey findings of 2013–2014 DRC DHS child participants aged 6–59 months [19], we also identified the high prevalence of seropositive individuals for type 1 and 2 in Kinshasa and type 3 in both Nord Kivu and Kinshasa. However, in contrast to the children’s results, adult DHS respondents showed a positive relationship between poliovirus immunity and the number of children residing in their households. This finding may again be suggestive of immunity resulting from infant care and fecal matter contact [10]. Behavioral transmission dynamics such as this are of particular importance for polio prevention and control through improved hygiene practices [23]. The correlation between serotypes, household size, and sex seen here all suggest that indirect vaccination by exposure to OPV shed from children may have contributed to immunity among adults in this survey.

While this study provides the first nationally representative estimates of poliovirus immunity among adults in the DRC, there are several limitations. As in the 2013–2014 child polio serosurvey, the adult serosurvey was carried out in conjunction with the household-based DHS, thus mobile or remote, difficult-to-reach populations may not be well represented in the sampling frame; indeed, there were no selected clusters near the previous cVDPV2 outbreak areas [19]. Despite efforts to split the adult specimens for even distribution across province, sex and age, and adjust survey weights for differential missingness, if other factors affecting serostatus were not evenly distributed across groups this may have affected the representativeness of the results. Another limitation is the lack of information on history of vaccination among adults, which was not collected by the DHS, other than tetanus for women who gave birth to one or more children. Given past SIAs that have included older children and adults, it would be of interest to assess seroprevalence in relation to this information. Finally, as noted above, we are unable to distinguish between immune responses induced by natural infection versus exposure to vaccine-related poliovirus in the laboratory analysis, a limitation of the neutralization assay [24].

## Conclusions

Our finding of high susceptibility to poliovirus among adults in the DRC is of concern, given the potential for importation of WPV1 or cVDPV. These results may have been influenced by several factors. While we found evidence for increased seropositivity with household size, and number of children in the household, secondary spread of vaccine viruses was not sufficient to achieve high immunity for all serotypes. Second, low seropositivity suggests more limited historical circulation of virus, potentially due to population distribution and rurality. Lastly, while it is generally believed that polio immunity is lifelong once acquired, waning immunity below the limit of detection in adulthood may be possible. Given the global effort to eradicate polio, this study provides a first step in understanding adult immunity against polioviruses in a setting with continued clusters of outbreaks; this may influence future vaccination policies countrywide.

## Supplementary Information

**Additional file 1: Table S1.** Comparison of DRC 2013–2014 adult DHS respondent demographics across sample split. **Table S2.** To test for intra-household correlation in seropositivity, we merged the adult and child datasets, and assessed whether any child and/or any adult in the household was seropositive for each type of polio. The table below summarizes the results across households. Using Pearson’s Chi-squared test, we find that sero-status has a mild positive association for Type 2 (p = 0.01), but there is little evidence for within household correlation for Types 1 and 3. **Figure S1.** Study design flowchart for dried blood spot (DBS) sampling, testing, and analysis. From a total of 8713 DBS collected in the field, 5526 (63.4%) were successfully processed and merged to DHS questionnaire data for inclusion in this analysis.

**Additional file 2. ** R code for data analysis.

**Additional file 3. ** Serology data for poliovirus among adults surveyed and sampled in the 2013-2014 DHS in the Democratic Republic of Congo.

## Data Availability

The DHS survey data generated and analyzed during the current study are publicly available upon dataset access registration approval at https://dhsprogram.com/data/new-user-registration.cfm. The laboratory data and corresponding code for analysis are included as supplementary files.

## Abbreviations

AFP: Acute flaccid paralysis
CDC: US Centers for Disease Control and Prevention
DBS: Dried blood spot
DHS: Demographic and Health Survey
DRC: Democratic Republic of the Congo
EPI: Expanded Program on Immunization
INRB: Institut National de Recherche Biomedicale (National Institute of Biomedical Research)
MMVRT: Measles, mumps, varicella zoster, rubella and tetanus
OPV: Oral polio vaccine
SIA: Supplemental immunization activity
VDPV: Vaccine-derived poliovirus
cVDPV: Circulating vaccine-derived poliovirus
VPD: Vaccine preventable disease
WPV: Wild poliovirus
