# Supplementary material for: Poliovirus immunity among adults in the Democratic Republic of the Congo: a cross-sectional serosurvey
Source: BMC Infect Dis. 2022 Jan 5;22:30. doi: 10.1186/s12879-021-06951-6 (PMC8728990; doi:10.1186/s12879-021-06951-6)
Supplement: Supplementary file 1 — Additional file 1: Table S1. Comparison of DRC 2013–2014 adult DHS respondent demographics across sample split. Table S2. To test for intra-household correlation in seropositivity, we merged the adult and child datasets, and assessed whether any child and/or any adult in the household was seropositive for each type of polio. The table below summarizes the results across households. Using Pearson’s Chi-squared test, we find that sero-status has a mild positive association for Type 2 (p = 0.01), but there is little evidence for within household correlation for Types 1 and 3. Figure S1. Study design flowchart for dried blood spot (DBS) sampling, testing, and analysis. From a total of 8713 DBS collected in the field, 5526 (63.4%) were successfully processed and merged to DHS questionnaire data for inclusion in this analysis. [file 12879_2021_6951_MOESM1_ESM.zip › Table S2.docx]

**Supplementary Table 2:** To test for intra-household correlation in seropositivity, we merged the adult and child datasets, and estimated the calculated whether any child and whether any adult in the household was seropositive for a type of polio. The table below summarizes the results across households. Using Pearson’s Chi-squared test, we find that sero-status has a mild positive association for Type 2 (p = 0.01), but there is little evidence for within household correlation for Types 1 and 3.

|  | | Type 1 | | Type 2 | | Type 3 | |
| --- | --- | --- | --- | --- | --- | --- | --- |
|  |  | Children in Household | | Children in Household | | Children in Household | |
|  |  | Any Positive | All Negative | Any Positive | All Negative | Any Positive | All negative |
| Adults in Household | Any Positive | 1376 | 228 | 1497 | 104 | 1012 | 279 |
|  | All Negative | 446 | 89 | 484 | 54 | 637 | 211 |
| p-value: | | 0.20 | | 0.01 | | 0.09 | |
