# Supplementary material for: Poliovirus immunity among adults in the Democratic Republic of the Congo: a cross-sectional serosurvey
Source: BMC Infect Dis. 2022 Jan 5;22:30. doi: 10.1186/s12879-021-06951-6 (PMC8728990; doi:10.1186/s12879-021-06951-6)
Supplement: Supplementary file 2 — Additional file 2. R code for data analysis. [file 12879_2021_6951_MOESM2_ESM.docx]

library(tidyverse)

library(haven)

library(survey)

library(scales)

options( survey.lonely.psu = "adjust" )

#read in PR data

pr = read_dta("CDPR61FL.zip")

pr = pr %>% mutate(across(where(is.labelled),as_factor))

#calculate number of children in each HH, in each age band

#used to estimate # under-15 in the household

children_table = pr %>%

group_by(hv001,hv002) %>%

summarise(children_0_5_calc = sum(hv105 %in% 0:5,na.rm=T),

children_0_15_calc = sum(hv105 %in% 0:14,na.rm=T)

)

pr = left_join(pr,children_table)

#subset to eligible adults:

pr_sub = pr %>% filter(hv027 =="men's survey", #male survey

hv117 == 'eligible' | #eligible for women's or mens' survey

hv118 == 'eligible'

)

#read in serology

sero = read_csv("adult_polio_serology.csv")

#na samples without sufficient sample, and calculate seropositive:

sero = sero %>% mutate(

Sabin_1 = if_else(!is.na(S1_comment),NA_real_,Sabin_1),

Sabin_2 = if_else(!is.na(S2_comment),NA_real_,Sabin_2),

Sabin_3 = if_else(!is.na(S3_comment),NA_real_,Sabin_3),

p1 = as.numeric(Sabin_1 >=3),

p2 = as.numeric(Sabin_2 >=3),

p3 = as.numeric(Sabin_3 >=3)

)

#merge data together

data = left_join(pr_sub,sero,by = c('hv001','hv002','hvidx') )

#rename data for convenience

data = data %>%

rename(cluster = hv001,

hhnum = hv002,

psu = hv021,

strata= hv022,

age = hv105,

sex = hv104,

residence = hv025,

province = hv024,

education = hv106,

wealth = hv270,

weight = hv028,

children05 = hv014,

province_new = shnprovin)

#note calculated under-5 != reported under-5 children in HH for some houses

data %>% filter(children05 != children_0_5_calc) %>% distinct(cluster,hhnum,.keep_all = T)

data = data %>% mutate(weight = weight/1e6,

age = as.numeric(age),

age_cat = cut(age,c(0,19,24,29,34,39,44,100),labels = c('15-19','20-24','25-29','30-34','35-39','40-44','44+')),

child_cat = cut(children05,c(-1,0,1,2,3,Inf),labels=c('None','1','2','3','4+')),

child15_cat = cut(children_0_15_calc,c(-1,0,2,5,Inf),labels=c('None','1-2','3-5','5+')))

# Tell R about the survey design, and subset to complete observations:

des = svydesign(ids = ~psu+hhnum, strata = ~strata, weight = ~weight, nest = TRUE, data = data)

des_complete = subset(des,!is.na(p1),!is.na(p2),is.na(p3))

#### raking to match province, rural/urban, and age weighting of the full sample #################

pop.province = data %>%

group_by(province_new) %>% summarise(Freq= sum(weight))

pop.residence = data %>%

group_by(residence) %>% summarise(Freq = sum(weight))

pop.age = data %>%

group_by(age_cat) %>% summarise(Freq= sum(weight))

pop.sex = data %>%

group_by(sex) %>% summarise(Freq= sum(weight))

des_ps = rake(des_complete,sample.margins = list(~province_new,~residence,~age_cat,~sex),population.margins = list(pop.province,pop.residence,pop.age,pop.sex))

#helper function to format svyby() output:

reshape_svyby = function(x){

x %>% unite(sabin1,matches('p1')) %>% unite(sabin2,matches('p2')) %>% unite(sabin3,matches('p3')) %>%

gather(serotype,tmp, matches('sabin')) %>%

separate(tmp,c('seroprevalence','lower','upper'),'_') %>%

mutate_at(vars(seroprevalence,lower,upper), funs(as.numeric)) %>%

mutate(serotype = recode(serotype, 'sabin1' = 'Type 1', 'sabin2' = 'Type 2', 'sabin3' = 'Type 3'))

}

#overall seroprevalence

overall =svymean(~p1+p2+p3,des_ps,na.rm=T,na.rm.all = T)

overall = as_tibble(overall) %>% mutate(serotype = c('Type 1','Type 2','Type 3')) %>%

bind_cols(confint(overall) %>% as_tibble %>% setNames(c('lower','upper'))) %>%

rename(seroprevalence=mean)

#seroprevalece by province

tab = svyby(~p1+p2+p3,

~province_new,

des,

FUN = svymean,na.rm.by = T,na.rm=T,na.rm.all = T,vartype = 'ci')

tab = reshape_svyby(tab)

tab %>% bind_rows(overall %>% mutate(province_new = 'overall')) %>%

mutate(Province = reorder(str_to_title(province_new),-seroprevalence*as.numeric(serotype=='Type 1') - 1000*as.numeric(province_new == 'overall'))) %>%

ggplot(aes(x=Province,y=seroprevalence,ymin = lower,ymax=upper, colour = serotype)) +

geom_pointrange(position = position_dodge(width = 0.5)) +

scale_y_continuous('Seroprevalence', labels=percent_format(), limits = c(0,1),breaks =(0:10)/10,oob = squish) +

scale_colour_discrete("Serotype") +

scale_x_discrete(NULL)+

theme_bw() +

theme(axis.text.x = element_text(angle = 45, hjust = 1),panel.grid.major.y = element_line(), panel.grid.minor.y = element_line(linetype = 2))

ggsave("Figure_1.png",width = 7,height=6,units='in',dpi=600)

# seroprevalence by sex and age group

tab_age_sex = svyby(~p1+p2+p3,~age_cat+sex,des_ps,FUN = svymean,na.rm.by = T,na.rm=T,na.rm.all = T,vartype = 'ci')

tab_age_sex = reshape_svyby(tab_age_sex)

tab_age_sex= tab_age_sex %>% mutate(sex = recode(sex,'male'='M','female'='F'))

tab_age_sex %>%

ggplot(aes(x=sex,y=seroprevalence,ymin = lower,ymax=upper, colour= serotype)) +

geom_pointrange(position = position_dodge(width = 0.3)) +

scale_y_continuous('Seroprevalence', labels=percent_format(), limits = c(0,1),breaks = c(0,.2,.4,.6,.8,1)) +

scale_colour_discrete("Serotype") +

scale_x_discrete(NULL)+

theme_bw() +

facet_wrap(~age_cat,nrow=1,scales = 'free_x')+

theme(panel.grid.major.y = element_line(), panel.grid.minor.y = element_line(linetype = 2))

ggsave("Figure_2.png",width = 6,height=4,units='in',dpi=600)

# Pattern of seroprevalence, among all adults, and by sex:

mysvymean = function(formula,design,...){

m = svymean(formula,design,na.rm=T)

ci = confint(m)

tibble(...,seroprevalence = as.numeric(m),lower=ci[,1],upper=ci[,2])

}

tab_combo = bind_rows(

svyby(~p1+p2+p3, ~sex,des_ps,svymean,na.rm.by = T,na.rm=T,na.rm.all = T,vartype = 'ci') %>% reshape_svyby,

svyby(~I(p1*p2*p3), ~sex,des_ps,svymean,na.rm.by = T,na.rm=T,na.rm.all = T,vartype = 'ci') %>%

setNames(c('sex','seroprevalence','lower','upper')) %>% mutate(serotype = 'Types 1, 2, and 3'),

svyby(~I(p1*p2), ~sex,des_ps,svymean,na.rm.by = T,na.rm=T,na.rm.all = T,vartype = 'ci') %>%

setNames(c('sex','seroprevalence','lower','upper')) %>% mutate(serotype = 'Types 1 and 2'),

svyby(~I(p1*p3), ~sex,des_ps,svymean,na.rm.by = T,na.rm=T,na.rm.all = T,vartype = 'ci') %>%

setNames(c('sex','seroprevalence','lower','upper')) %>% mutate(serotype = 'Types 1 and 3'),

svyby(~I(p2*p3), ~sex,des_ps,svymean,na.rm.by = T,na.rm=T,na.rm.all = T,vartype = 'ci') %>%

setNames(c('sex','seroprevalence','lower','upper')) %>% mutate(serotype = 'Types 2 and 3'),

svyby(~I(p1*(1-p2)*(1-p3)), ~sex,des_ps,svymean,na.rm.by = T,na.rm=T,na.rm.all = T,vartype = 'ci') %>%

setNames(c('sex','seroprevalence','lower','upper')) %>% mutate(serotype = 'Type 1 only'),

svyby(~I(p2*(1-p1)*(1-p3)), ~sex,des_ps,svymean,na.rm.by = T,na.rm=T,na.rm.all = T,vartype = 'ci') %>%

setNames(c('sex','seroprevalence','lower','upper')) %>% mutate(serotype = 'Type 2 only'),

svyby(~I(p3*(1-p1)*(1-p2)), ~sex,des_ps,svymean,na.rm.by = T,na.rm=T,na.rm.all = T,vartype = 'ci')%>%

setNames(c('sex','seroprevalence','lower','upper')) %>% mutate(serotype = 'Type 3 only'),

svyby(~I((1-p3)*(1-p1)*(1-p2)), ~sex,des_ps,svymean,na.rm.by = T,na.rm=T,na.rm.all = T,vartype = 'ci')%>%

setNames(c('sex','seroprevalence','lower','upper')) %>% mutate(serotype = 'All negative'),

mysvymean(~p1+p2+p3, des_ps,sex='All adults',serotype = c('Type 1','Type 2','Type 3')),

mysvymean(~I(p1*p2*p3), des_ps,sex='All adults',serotype = 'Types 1, 2, and 3'),

mysvymean(~I(p1*p2), des_ps,sex='All adults',serotype = 'Types 1 and 2'),

mysvymean(~I(p1*p3), des_ps,sex='All adults',serotype = 'Types 1 and 3'),

mysvymean(~I(p2*p3), des_ps,sex='All adults',serotype = 'Types 2 and 3'),

mysvymean(~I(p1*(1-p2)*(1-p3)), des_ps,sex='All adults',serotype = 'Type 1 only'),

mysvymean(~I(p2*(1-p1)*(1-p3)), des_ps,sex='All adults',serotype = 'Type 2 only'),

mysvymean(~I(p3*(1-p1)*(1-p2)), des_ps,sex='All adults',serotype = 'Type 3 only'),

mysvymean(~I((1-p3)*(1-p1)*(1-p2)), des_ps,sex='All adults',serotype = 'All negative')

)

tab_combo_display = tab_combo %>% mutate(across(c(seroprevalence,lower,upper),~100*round(.,2))) %>%

transmute(sex,serotype,values = str_c(seroprevalence, " (",lower,'-',upper,')'))

tab_combo_display = tab_combo_display %>% pivot_wider(values_from=values,names_from = sex) %>%

select(serotype,`All adults`,Male = male,Female = female)

tab_combo_display

tab_combo_display %>% write_csv('table_1.csv')

#seroprevalence by demographics:

tab_demos = bind_rows(

svyby(~p1+p2+p3, ~sex,des_ps,svymean,na.rm.by = T,na.rm=T,na.rm.all = T,vartype = 'ci') %>% reshape_svyby %>%

rename(level=sex) %>% mutate(variable = 'Sex'),

svyby(~p1+p2+p3, ~age_cat,des_ps,svymean,na.rm.by = T,na.rm=T,na.rm.all = T,vartype = 'ci') %>% reshape_svyby %>%

rename(level=age_cat) %>% mutate(variable = 'Age'),

svyby(~p1+p2+p3, ~education,des_ps,svymean,na.rm.by = T,na.rm=T,na.rm.all = T,vartype = 'ci') %>% reshape_svyby %>%

rename(level=education) %>% mutate(variable = 'Education'),

svyby(~p1+p2+p3, ~wealth,des_ps,svymean,na.rm.by = T,na.rm=T,na.rm.all = T,vartype = 'ci') %>% reshape_svyby %>%

rename(level=wealth) %>% mutate(variable = 'Wealth'),

svyby(~p1+p2+p3, ~child_cat,des_ps,svymean,na.rm.by = T,na.rm=T,na.rm.all = T,vartype = 'ci') %>% reshape_svyby %>%

rename(level=child_cat) %>% mutate(variable = 'Children 0-5'),

svyby(~p1+p2+p3, ~child15_cat,des_ps,svymean,na.rm.by = T,na.rm=T,na.rm.all = T,vartype = 'ci') %>% reshape_svyby %>%

rename(level=child15_cat) %>% mutate(variable = 'Children 0-14'),

svyby(~p1+p2+p3, ~residence,des_ps,svymean,na.rm.by = T,na.rm=T,na.rm.all = T,vartype = 'ci') %>% reshape_svyby %>%

rename(level=residence) %>% mutate(variable = 'Type of Residence')

)

#reformat for pretty printing:

tab_demos_display = tab_demos %>% mutate(across(c(seroprevalence,lower,upper),~100*round(.,2))) %>%

transmute(variable,level,serotype,values = str_c(seroprevalence, " (",lower,'-',upper,')'))

tab_demos_display = tab_demos_display %>% pivot_wider(names_from=serotype,values_from=values)

#calculate population demographics for reference:

demos_proportion = svymean(~sex+age_cat+wealth+education+child_cat+child15_cat+residence,des,na.rm=T) %>%

as_tibble(rownames = 'level') %>%

mutate(variable = str_extract(level,'sex|age_cat|wealth|education|child_cat|child15_cat|residence'),

level = str_replace(level,'sex|age_cat|wealth|education|child_cat|child15_cat|residence',''),

variable = recode(variable,sex='Sex',wealth = 'Wealth',age_cat='Age',education = 'Education',child_cat='Children 0-5',child15_cat='Children 0-14',residence='Type of Residence')) %>%

transmute(variable,level,Proportion = 100*round(mean,2))

tab_demos_display = left_join(tab_demos_display,demos_proportion) %>%

relocate(variable,level,Proportion)

#perform tests:

## updating design to fix error in svychisq() when empty factor levels in education

des_ps = update(des_ps,education2 = as.character(education))

test_df = expand_grid(serotype = c('p1','p2','p3'),

variable = c('sex','age_cat','child_cat','child15_cat','wealth','education2','residence')) %>%

mutate(formula = str_c('~',serotype,'+',variable))

test_df = test_df %>% rowwise() %>%

mutate(p.value = svychisq(as.formula(formula),des_ps,na.rm=T)$p.value)

test_df = test_df %>% mutate(serotype = recode(serotype,'p1'='Type 1','p2' = 'Type 2','p3' = 'Type 3'),

variable = recode(variable,sex='Sex',wealth = 'Wealth',age_cat='Age',education2 = 'Education',child15_cat='Children 0-14',child_cat='Children 0-5',residence='Type of Residence'))

test_df = test_df %>% mutate(p.value = str_c('p=',format(p.value,digits=2))) %>% select(-formula) %>%

pivot_wider(names_from = serotype,values_from = p.value)

tab_demos_display = bind_rows(tab_demos_display,test_df)

tab_demos_display = tab_demos_display %>%

arrange(variable)

tab_demos_display

write_csv(tab_demos_display,'table_2.csv')
